# Orthopedic implant allergies: a review of current knowledge on metal hypersensitivity and prosthesis failure

**DOI:** 10.1530/EOR-2025-0252

**Published:** 2026-10-01

**Authors:** Veronica Viola, Niccoló Marelli, Teofila Caplanusi, Sylvain Steinmetz

**Affiliations:** ^1^ Faculty of Biology and Medicine, University of Lausanne, Lausanne, Switzerland; ^2^ Department of Orthopedics and Traumatology, Regional Hospital Bellinzona e Valli, Ente Ospedaliero Cantonale, Bellinzona, Switzerland; ^3^ Department of Dermatology, Lausanne University Hospital and University of Lausanne, Lausanne, Switzerland; ^4^ Department of Orthopedics and Traumatology, Lausanne University Hospital and University of Lausanne, Lausanne, Switzerland

**Keywords:** allergy, prosthesis, hypersensitivity, adverse effect, arthroplasty, revision, metals

## Abstract

- Metal hypersensitivity is an increasingly recognized factor in orthopedic implant failure, particularly in patients undergoing total joint arthroplasty (TJA), and may contribute to persistent pain, aseptic loosening, or early revision.
- This review synthesizes current evidence on the underlying immune mechanisms of metal hypersensitivity and explores their potential role in implant-related complications such as chronic inflammation, pain, and aseptic loosening.
- A systematic literature search was conducted using Embase.com and complementary databases; relevant studies involving patients with suspected or confirmed allergic responses to metallic orthopedic implants were identified and integrated into a narrative review of clinical presentations, diagnostic strategies, and outcomes.
- Diagnostic approaches remain variable and include clinical evaluation, dermatological patch testing, *in vitro* assays such as the lymphocyte transformation test (LTT), and histopathological examination of periprosthetic tissues, with no current consensus on a gold-standard method.
- Although metal hypersensitivity is associated with a subset of implant failures, the strength of evidence is limited by heterogeneous study designs, variable diagnostic criteria, and inconsistent reporting of outcomes.
- Future efforts should focus on standardizing diagnostic protocols, validating predictive tests, and developing patient-specific management algorithms – particularly regarding implant material selection – to improve care for patients with suspected metal allergies in orthopedic surgery.

## Introduction

Total joint arthroplasty (TJA) is a widely performed procedure for managing end-stage osteoarthritis, significantly improving pain relief and mobility in affected patients. However, complications such as chronic pain, swelling, dermatitis, and impaired wound healing remain major concerns (1, 2). In addition, metal hypersensitivity has increasingly been identified as a potential cause of implant-related adverse reactions (3). Despite advances in the field of prosthetics, reports suggest that a subset of patients develop local symptoms unexplained by infection, aseptic loosening, mechanical failure, or malalignment (4, 5).

Metal hypersensitivity, primarily mediated by type IV delayed hypersensitivity reactions, involves immune system sensitization to metallic ions released from implant materials, such as cobalt, chromium, nickel, titanium, and stainless steel (6). The release of these ions occurs through corrosion, mechanical wear, or tribocorrosion processes at the implant–tissue interface. The body’s immune response is characterized by an inflammatory cascade, mediated by activated T lymphocytes and macrophages, resulting in chronic local inflammation and osteolysis, ultimately leading to implant loosening and failure (7).

This hypersensitivity can lead to chronic inflammation, damage to surrounding tissue, and eventual implant failure (8). The most commonly implicated metals in orthopedic devices include nickel, cobalt, and chromium, which are frequently found in cobalt–chromium alloy and stainless-steel implants (9). Although titanium- and zirconium-based implants are generally considered hypoallergenic, rare cases of hypersensitivity have been reported (10).

Clinical manifestations of metal hypersensitivity can vary noticeably, ranging from localized dermatitis to severe implant loosening and periprosthetic osteolysis. Patients may present with unexplained pain, persistent joint effusion, or skin rashes, while others experience progressive implant instability without clear clinical indicators (11, 12).

Diagnosing metal hypersensitivity is challenging, as symptoms frequently overlap with other causes of implant failure (13).

In metal-on-metal hip resurfacing and large-diameter total hip arthroplasty, adverse reactions to metal debris (ARMD) have also been described, including pseudotumor formation, soft-tissue destruction, and early implant failure, particularly in association with elevated cobalt and chromium levels and increased component wear (14).

Several studies have aimed to determine the prevalence of metal hypersensitivity in TJA recipients, yet reported rates vary widely due to differing diagnostic criteria, patient populations, and testing methodologies (15). Patch testing, lymphocyte transformation tests (LTT), and histological examination of periprosthetic tissue have all been employed to identify affected patients, although no definitive diagnostic standard has yet emerged. Consequently, therapeutic strategies remain controversial, ranging from conservative symptomatic treatment to revision surgery employing hypoallergenic implants (16, 17).

This review aims to provide an exhaustive analysis of metal hypersensitivity in orthopedic implants by summarizing the current understanding of its pathophysiology, diagnostic challenges, clinical consequences, and management strategies. Through a review of the current literature, we aim to highlight gaps in knowledge and identify where further research is needed to eventually improve patient outcomes following TJA.

## Methods

The review was initially conceived as a systematic review; however, owing to the insufficient availability of well-controlled studies suitable for quantitative pooling, the analysis was conducted through a narrative and qualitative synthesis. The study has been registered with the PROSPERO database (registration number: CRD42023408652).

A structured literature search was performed using Embase.com, Medline Ovid ALL, Cochrane Library Wiley, CENTRAL – Cochrane Library Wiley, Web of Science – Core collection, Proquest D&T, and Google Scholar, ensuring extensive coverage of the topic. The search strategy included both indexed terms and free-text terms related to orthopedic prostheses, metal allergies, hypersensitivity, and revision surgeries. The research strategy was the following:

(‘Orthopedic prostheses, orthoses and implants’/exp OR ‘osteosynthesis material*’:ab,ti,kw OR orthopedic*:ab,ti,kw OR orthopedic*:ab,ti,kw OR (((implant OR implants OR implanted) NEAR/3 (ortho* OR metal* OR revision* OR surgical OR knee* OR hip OR hips OR ankle*)):ab,ti,kw) OR ‘joint replacement*’:ab,ti,kw OR biomaterial*:ab,ti,kw OR (((surger* OR arthroplasty*) NEAR/3 (replacement OR knee* OR hip OR hips OR ankle)):ab,ti,kw) OR prosthes*:ab,ti,kw) AND (((‘hypersensitivity’/exp OR ‘hypersensitivity concepts’/exp OR ‘prosthesis complication’/de OR ‘prosthesis loosening’/exp) AND ‘metal’/exp) OR ‘hypersensitivity’/de OR ‘allergic disease’/de OR ‘allergic reaction’/de OR ‘allergy’/de OR ‘delayed hypersensitivity’/exp OR (((hypersensitiv* OR sensitiv* OR allerg* OR atopy OR atopic OR urticaria OR erythem* OR reaction*) NEAR/6 (metal* OR alloy* OR aluminium OR aluminum OR chromium OR cobalt OR iron OR ferrum OR ferro OR nickel OR titan* OR vanadium)):ab,ti,kw)) NOT (‘conference abstract’/it OR ‘conference review’/it).

Studies included in this review met the following inclusion criteria:

- Studies reporting patients with suspected or confirmed allergic reactions to metallic prosthetic joints (knees, hips, ankles, shoulders, and elbows).
- Studies reporting patients who underwent revision surgery due to suspected or confirmed metal hypersensitivity.
- Studies reporting participant symptoms, type of prosthesis, surgical details, and postoperative outcomes.

Exclusion criteria included:

- Studies focusing on other types of metallic implants or non-orthopedic implants.
- Non-human studies.
- Conference abstracts, literature reviews without original data, case reports and case series.

The selection process followed PRISMA guidelines. The initial search across all databases retrieved 2,714 abstracts. These were first screened for relevance by a single reviewer based on title and abstract. From this step, 182 studies were considered potentially eligible, and their full texts were retrieved.

Full-text articles were then independently assessed by two reviewers according to the predefined inclusion and exclusion criteria. In cases where the reviewers disagreed, discrepancies were resolved through discussion until a consensus was reached.

This multi-step approach ensured rigorous selection and minimized the risk of bias (Fig. 1).

**Figure 1:**
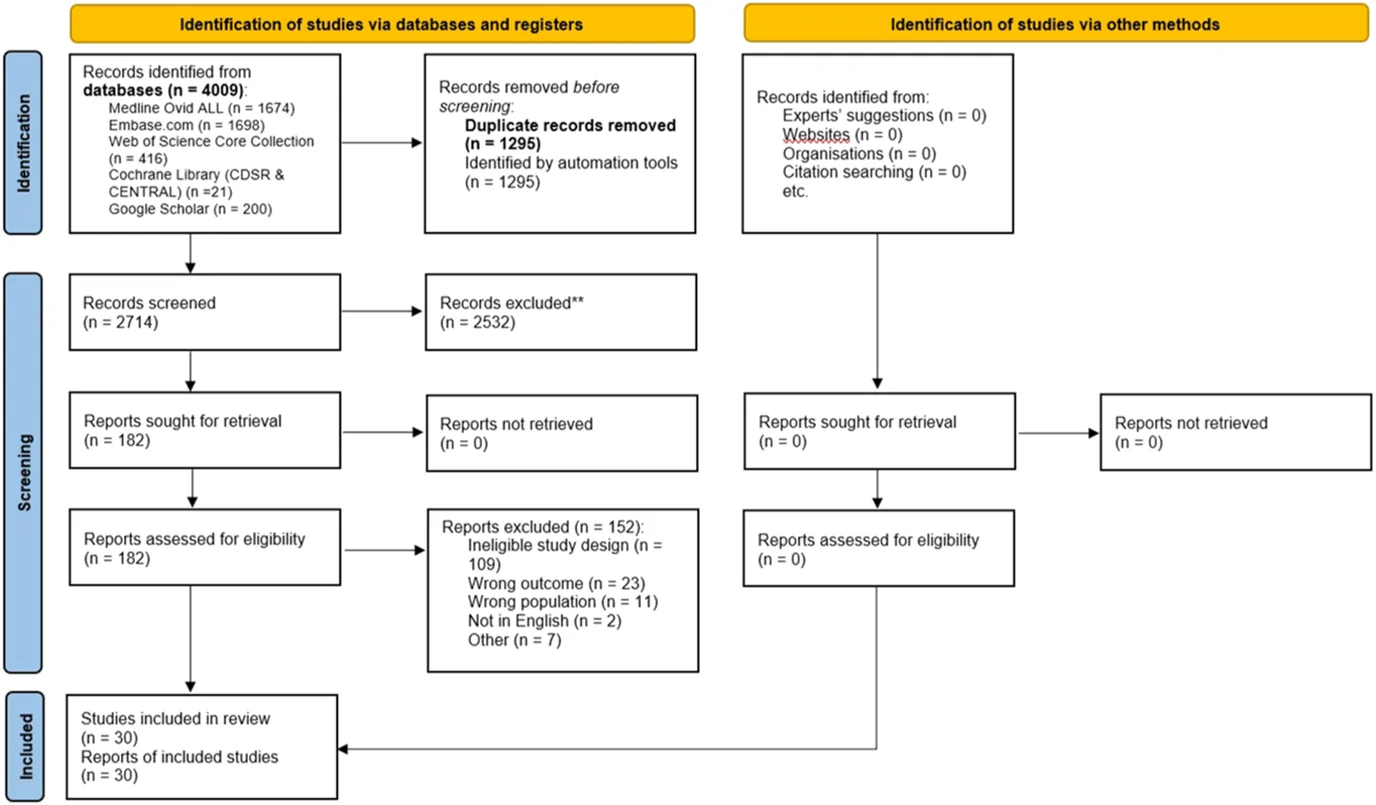
Overview of the identification, screening, and inclusion process, resulting in 30 studies retained for qualitative synthesis.

In addition to the studies included in the systematic analysis, several relevant publications were consulted to support the background and pathophysiological discussion. These articles, including studies suggested during peer review, were cited as contextual references but were not included in the systematic review dataset.

Data were extracted by one reviewer and verified for accuracy during manuscript preparation, focusing on study design, patient population, diagnostic methods, types of prosthesis, surgical details, and clinical outcomes. The lack of consistent and reliable statistical data across studies made it impossible to conduct a meta-analysis. Instead, a qualitative synthesis of the evidence was performed to identify patterns, gaps, and inconsistencies in the literature.

## Results

A total of 30 studies were included in this review, comprising a wide range of study designs including revision outcome reports (*n* = 9), observational studies (*n* = 8), histological or diagnostic testing evaluations (*n* = 8), and registry-based or review articles (*n* = 5). The sample sizes of individual studies varied significantly, ranging from 0 to 3,275 patients, with an average of approximately 263 patients per study.

### Study outcomes

Of the 30 studies reviewed, 5 (17%) reported clinical improvement following revision surgery with hypoallergenic implants such as titanium or ceramic components in patients with suspected or confirmed metal hypersensitivity (9, 13, 15, 18, 19).

These studies, mostly small case series, described symptom resolution rates of up to 100% following revision for allergy-related implant failure, particularly in cases of confirmed nickel or cobalt sensitization.

In contrast, two studies (7%) evaluating revision procedures in patients with suspected allergy reported no significant improvement in functional or symptomatic outcomes (20, 21). These findings suggest that, although the use of hypoallergenic components may reduce immunogenic exposure, this does not systematically translate into clinical benefit.

Three studies (10%) documented persistently poor outcomes despite revision, including continued pain, radiographic loosening, or re-revision in patients with suspected hypersensitivity (2, 6, 22).

These findings emphasize the obstacles to reliably diagnosing metal allergy and underscore that revision does not guarantee symptom resolution.

An additional two studies (7%) presented inconclusive or mixed outcomes following revision, typically limited by small sample sizes or variable diagnostic protocols (1, 10).

Beyond revision-focused literature, six studies (20%) evaluated primary implantation using hypoallergenic components in patients with known or suspected allergy (8, 9, 13, 23, 24, 25).

These studies generally reported good outcomes, although their preventive nature and absence of control groups limit conclusions regarding the clinical benefit of coated or ceramic implants in allergic patients.

Three studies (10%) compared primary arthroplasty outcomes between allergic and non-allergic patients and found no significant difference in functional results or complication rates (26, 27, 28).

Three older case series (10%) provided anecdotal or historical insights suggesting a potential relationship between metal sensitivity and implant failure, but lacked systematic diagnostics or follow-up (3, 4, 29).

One study (3%) focused exclusively on diagnostic testing, evaluating patch testing and lymphocyte transformation testing without linking findings to clinical outcomes (6).

Finally, five studies (17%) were review articles (17, 30), registry-based studies, or investigations lacking longitudinal postoperative outcome data. Some of these included assessments of patient-reported symptoms such as pain, alongside diagnostic or histological findings (31, 32, 33), but did not report on treatment responses or clinical improvement following interventions, and were thus considered as providing background context rather than evaluable outcome data.

### Implant materials and reported outcomes

Studies involving hypoallergenic materials, such as titanium, ceramic, or oxinium, were more frequently associated with positive outcomes when used in the context of revision surgery for suspected metal hypersensitivity. All five studies reporting improvement after revision used such materials (9, 13, 15, 18, 19).

By contrast, in primary arthroplasty settings, six studies evaluated the use of hypoallergenic implants in patients with self-reported or diagnosed metal allergy (10, 11, 16, 23, 24, 25). Although outcomes were generally favorable, these studies were preventive in nature and did not include revision or symptomatic reversal. Their observational design limits conclusions about the therapeutic effect of hypoallergenic materials.

Furthermore, three studies comparing standard versus coated implants or allergic versus non-allergic patients after primary arthroplasty found no significant differences in outcomes, suggesting that the presence of self-reported allergy may not always influence implant performance (26, 27, 28).

### Functional and symptomatic measures

Only a minority of studies employed validated scoring systems to assess postoperative function, and their findings were heterogeneous:

- Nam *et al.* (21) found that patients with self-reported metal allergy had significantly lower Knee Society Function Scores and lower satisfaction levels.
- Schmidt *et al.* (28) observed significantly reduced KSS and SF-12 Physical Component scores in allergic patients compared with controls.
- Ko *et al.* (15) reported complete symptom resolution following shoulder arthroplasty revision in allergic patients.

Most other studies reporting symptomatic or functional improvement relied on subjective reporting, without standardized metrics, limiting direct comparability.

### Diagnostic variability

Diagnostic approaches across studies included epicutaneous patch testing, lymphocyte transformation testing (LTT), and histological analysis of periprosthetic membranes. Most authors acknowledged that no single diagnostic modality is sufficient to reliably confirm metal hypersensitivity in the orthopedic context. Among the eight studies incorporating diagnostic strategies, four supported a multimodal approach, combining clinical evaluation, immunological testing, and histological analysis to strengthen diagnostic certainty.

However, several studies raised concerns regarding the limited specificity, poor reproducibility, and lack of standardization associated with patch testing and LTT. The clinical relevance of positive test results also remained controversial in the absence of consistent correlation with symptoms or implant performance. One study focused exclusively on diagnostic evaluation without reporting patient outcomes, emphasizing the need for further clinical validation (8).

### Studies reporting diagnostic and histological findings without longitudinal outcomes

Three studies in this review provided important diagnostic and histological insights related to metal hypersensitivity in arthroplasty patients, though none reported postoperative outcomes or intervention effects.

- Caicedo *et al.* (31) assessed metal sensitivities in 23 patients presenting with postoperative pain after total joint arthroplasty using multi-metal lymphocyte transformation testing (LTT). They found that 48% of symptomatic patients exhibited metal sensitivity, supporting LTT as a potentially valuable diagnostic adjunct in this population.
- Willert *et al.* (32) performed detailed histomorphological analysis of periprosthetic tissues from patients with metal-on-metal hip implants. They documented features consistent with adverse local tissue reactions (ALVAL), such as lymphocytic infiltration and tissue necrosis, highlighting the immunological basis of hypersensitivity reactions without correlating findings to clinical outcomes.
- Milavec-Puretic *et al.* (33) evaluated patch test positivity to metals in 40 patients with failed hip endoprostheses, finding a high prevalence of sensitivity to cobalt and nickel. However, no subsequent clinical follow-up was reported to assess the impact of these sensitivities on implant survival or symptom resolution.

These studies provide precious diagnostic and pathological context to metal hypersensitivity but highlight the need for further prospective research linking such findings to clinical outcomes and treatment effectiveness.

### Quantitative trends

- 17% (*n* = 5) of studies reported improvement following revision with hypoallergenic implants.
- 7% (*n* = 2) found no improvement after revision.
- 10% (*n* = 3) reported worsened or persistently poor outcomes despite revision.
- 7% (*n* = 2) presented inconclusive or mixed results following revision.
- 20% (*n* = 6) examined outcomes after primary implantation of hypoallergenic prosthesis in allergic patients.
- 10% (*n* = 3) compared outcomes by allergy status following primary arthroplasty and found no significant difference.
- 10% (*n* = 3) were historical or anecdotal reports lacking standardized methodology.
- 3% (*n* = 1) focused solely on diagnostic testing without clinical follow-up.
- 17% (*n* = 5) were review or registry-based articles without original clinical data.

## Pathophysiology of metal hypersensitivity

Metal hypersensitivity is a delayed-type hypersensitivity (type IV immune reaction) triggered by metal ions released from implants.

In addition to articular wear, corrosion at modular junctions represents an important source of metal release. Meyer *et al.* (34) reported that in 114 revisions of large-diameter metal-on-metal total hip arthroplasties, 94% showed instability at the cone–taper interface, with associated galvanic corrosion, abundant titanium and iron release, and predominantly macrophage-driven foreign-body reactions with tissue necrosis. These findings support the concept that mechanically assisted crevice or fretting corrosion may generate metal debris capable of initiating inflammatory and potentially hypersensitivity-related periprosthetic reactions.

These ions bind to native proteins, forming haptens that elicit an immune response. Sensitization leads to a T-cell-mediated reaction, resulting in chronic inflammation and potential implant failure (17, 27, 33). Histological findings in hypersensitive patients often reveal perivascular lymphocytic infiltrates, macrophage accumulation, and evidence of tissue necrosis. Factors influencing the risk of hypersensitivity include genetic predisposition, pre-existing cutaneous metal allergies, and implant composition (18, 25) (Fig. 2).

**Figure 2:**
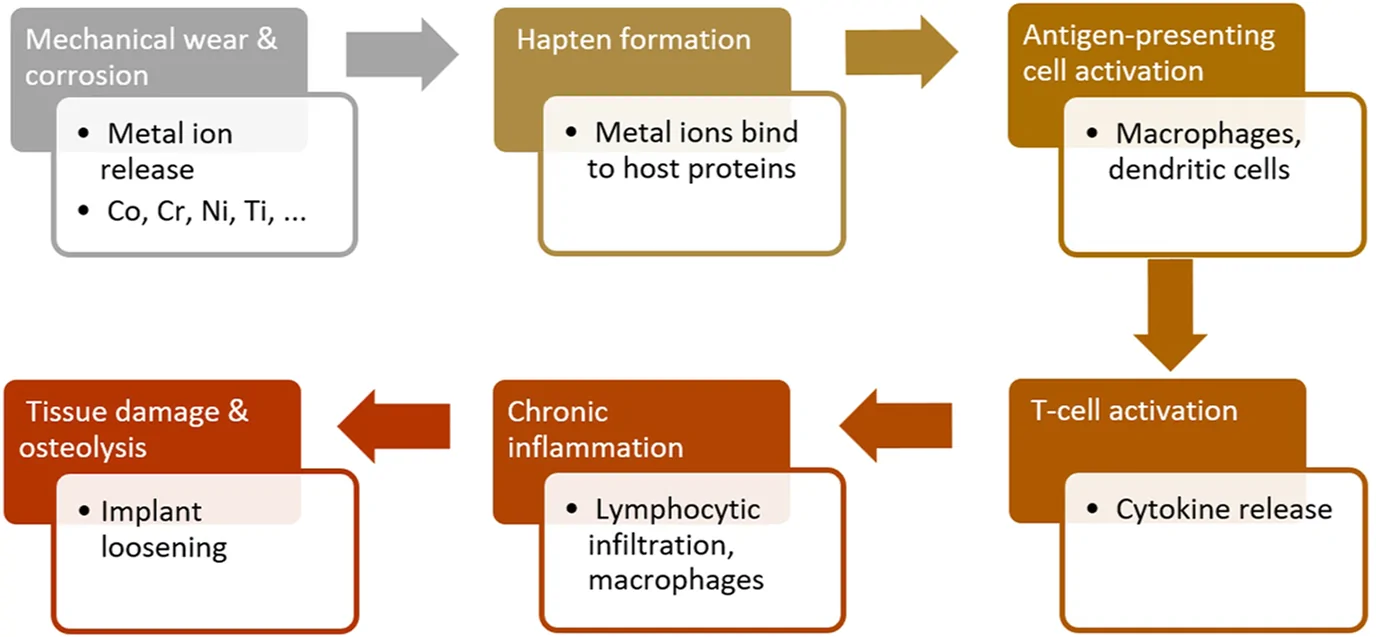
Mechanical wear and corrosion lead to the release of metal ions, which bind to host proteins forming haptens. These complexes activate antigen-presenting cells, resulting in T-cell activation and cytokine release. The ensuing chronic inflammation, characterized by lymphocytic infiltration and macrophage activity, contributes to tissue damage, osteolysis, and ultimately implant loosening.

The biological response to implant-derived particles is also mediated by antigen-presenting and phagocytic cells within the periprosthetic membrane. Chamaon *et al.* (35) demonstrated expression of the dendritic-cell marker CD11c in revision total hip arthroplasty tissues, whereas control capsular tissues obtained during primary arthroplasty were negative. CD11c-positive cells were observed in several macrophage- and giant-cell-like patterns and were particularly associated with UHMWPE-containing tissues, suggesting that antigen presentation and macrophage-lineage immune activation may contribute to the local inflammatory response to implant debris.

Current research focuses on hypoallergenic implants with reduced metal ion release and improved biocompatibility. Emerging surface modifications, such as ceramic coatings and alternative alloy compositions, demonstrate potential to reduce hypersensitivity reactions. However, further studies are needed to determine their long-term efficacy (10, 26).

## Incidence and clinical presentation

The prevalence of metal hypersensitivity in the general population is estimated to be between 10 and 15%, with orthopedic implant recipients demonstrating similar or slightly elevated rates due to chronic exposure to metallic debris. Studies have reported that up to 25% of patients having patch testing prior to TJA show sensitivity to at least one metal, with nickel being the most common allergen. However, the clinical relevance of pre-existing metal allergies in implant recipients remains controversial, as many patients with documented hypersensitivity do not develop complications (1, 8, 27, 33).

Symptoms of metal hypersensitivity can emerge weeks to months after implantation and may include localized pain, swelling, pruritus, and dermatitis over the surgical site. In more severe cases, progressive aseptic loosening, persistent joint effusions, and osteolysis may necessitate early revision surgery.

In the specific context of metal-on-metal resurfacing, Langton and colleagues (14, 36) reported that ARMD-related failures were associated with elevated blood and joint cobalt/chromium concentrations, increased component wear, and, in some designs, substantially higher mid-term failure rates than comparator resurfacing systems.

Differentiating these symptoms from mechanical failure, infection, or other inflammatory conditions can be challenging, which frequently results in delays in diagnosis and treatment (2, 9, 18). The latest research stresses the importance of adopting uniform diagnostic protocols to accurately identify patients at risk of metal hypersensitivity-related implant failure (17).

### Impact on prosthesis survival and revision rates

Several studies have reported increased revision rates among patients with suspected metal hypersensitivity. Implant failure due to hypersensitivity is often characterized by aseptic loosening, peri-implant osteolysis, and persistent pain (21, 24).

Registry data suggest that patients undergoing revision for suspected metal allergy may benefit from hypoallergenic implants, though long-term outcomes remain unclear (13).

A review of joint registries indicates that patients with self-reported metal allergies have an increased likelihood of undergoing early revision surgery compared with those without allergies, with poorer postoperative outcomes and lower patient satisfaction (21, 28). The revision burden associated with metal hypersensitivity remains significant, with studies reporting higher rates of implant failure and revision surgeries in patients with metal sensitization. Research indicates that metal sensitization is more common in failed total joint replacements, with some studies showing a prevalence of up to 60% in poorly functioning implants. In addition, registry data suggest that patients with multiple revisions have a higher prevalence of cobalt and chromium allergies, further supporting the potential link between metal hypersensitivity and implant failure (9, 24, 30).

Some reports suggest that patients with suspected metal allergy experience worse functional outcomes and lower postoperative satisfaction, particularly in TKA and THA. These patients report lower Knee Society Function Scores, symptoms, satisfaction, and expectation subdomains, highlighting the need to improve preoperative screening strategies (21, 28).

In cases of confirmed metal hypersensitivity, revision surgery using hypoallergenic materials, such as titanium-, ceramic-, or oxinium-coated implants, has demonstrated promising results. Patients undergoing revision with hypoallergenic materials report greater symptomatic relief and improved implant longevity (9, 10, 19). However, the long-term survivorship of these alternative materials remains under investigation, as some studies indicate an increased risk of wear and mechanical failure in certain ceramic and coated implants (23, 26).

Despite these findings, many orthopedic surgeons remain hesitant to attribute implant failure solely to metal hypersensitivity due to the lack of definitive diagnostic criteria. Future research should focus on the long-term follow-up of revision cases and the standardization of testing protocols to better identify at-risk patients (2, 17, 25).

## Diagnostic considerations

Diagnosing metal hypersensitivity in TJA patients is challenging, as no single test provides definitive confirmation. Patch testing, though commonly used for contact dermatitis, has variable reliability in implant-related hypersensitivity, due to its inability to assess deep-seated immune reactions, and it may produce false positives or negatives.

The lymphocyte transformation test (LTT) offers a functional assessment of T-cell reactivity to metal ions, but its high cost, lack of standardization, and insufficient validation limit its widespread clinical use (17, 30).

Histological analysis of periprosthetic tissue can reveal inflammatory changes suggestive of hypersensitivity, such as perivascular lymphocytic infiltrates, macrophage accumulation, and fibrosis; yet, these findings are not specific (Table 1).

**Table 1:** Comparative overview of diagnostic tests commonly used to evaluate metal hypersensitivity in patients with orthopedic implants. Each method presents specific advantages and limitations, and no single test currently serves as a definitive diagnostic standard.

Test	What it measures	Advantages	Limitations
Patch test	Cutaneous delayed-type reaction	Widely available, low cost	May give false positives; does not assess deep tissues
LTT	T-cell proliferation to metal ions	Quantitative, immune-specific	Expensive, limited labs, variable reproducibility
Histology	Tissue-level immune response	Confirms inflammation type	Invasive, post-surgery only

Histological analyses of failed metal-on-metal implants have demonstrated heterogeneous tissue responses ranging from macrophage-dominated foreign–body reactions and tissue necrosis to lymphocyte-rich inflammatory infiltrates characteristic of adverse reactions to metal debris (ARMD) (36, 37).

The histopathological classification of periprosthetic membranes was first proposed by Krenn *et al.* (38) to standardize the criteria for assessing implant-related complications. This original system includes four types:

- Type I (wear particle-induced), characterized by macrophages and multinucleated giant cells containing wear debris.
- Type II (infectious), marked by the presence of neutrophilic granulocytes without significant wear particles.
- Type III (combined), showing features of both type I and type II.
- Type IV (indeterminate), which does not meet the criteria for the previous types and typically shows fibrous tissue with minimal inflammation.

In 2014, the classification was expanded into the SLIM (synovial-like interface membrane) consensus classification, adding three new categories to cover a wider variety of pathological responses (39).

- Type V corresponds to arthrofibrosis, dominated by dense fibrous tissue with minimal inflammatory infiltrate.
- Type VI represents hypersensitivity reactions, showing perivascular lymphocytic infiltrates suggestive of an immune response to implant materials.
- Type VII includes toxic reactions, typically caused by corrosion products or metal ions, especially in metal-on-metal implants.

This expanded classification provides a more comprehensive diagnostic framework that helps guide clinical decision-making in cases of implant failure, including allergic and metal-related adverse tissue reactions. Most allergic reactions to orthopedic implants are classified as type VI in the expanded SLIM classification, characterized by perivascular lymphocytic infiltrates indicative of a delayed-type hypersensitivity response (38, 39).

Advanced imaging and serological biomarkers have been studied as possible diagnostic tools but require further validation. Current diagnostic approaches rely on a combination of patient history, clinical symptoms, and laboratory testing, as no single test can definitively confirm metal hypersensitivity (15, 19).

## Alternative approaches to patient management

### Hypoallergenic implant materials

Recent technological developments in prosthetics have led to the introduction of hypoallergenic alternatives, including ceramic-on-ceramic and ceramic-on-polyethylene implants. These implants have shown favorable outcomes while eliminating metal exposure, reducing hypersensitivity risks (17, 23).

Coated implants, such as titanium- or oxinium-coated components, appear promising in reducing metal ion release while maintaining mechanical strength. Studies confirm that TiN and ZrN coatings significantly decrease nickel, cobalt, and chromium ion release, reducing the risk of hypersensitivity reactions. Clinical studies report improved patient outcomes with these materials; however, further research is needed to assess long-term durability (10, 12, 26).

### Preoperative screening and risk assessment

Routine allergy testing before implant selection remains debated. Although some experts endorse the idea of preoperative patch testing in high-risk patients, others question its predictive value, and routine testing is not recommended. Personalized implant selection based on patient history and risk stratification may optimize outcomes. Current guidelines emphasize a patient-centered approach, incorporating shared decision-making and multidisciplinary evaluation.

### Revision surgery outcomes in metal-hypersensitive patients

Patients undergoing revision surgery for suspected metal hypersensitivity often report symptomatic relief following implant exchange. Studies indicate that revision with hypoallergenic materials can improve pain scores and functional outcomes. However, complications like bone loss and surgical morbidity remain concerns. Further studies are required to define standardized revision protocols for metal-sensitive patients.

## Discussion

This review indicates that metal hypersensitivity may contribute to adverse outcomes in a subset of patients with orthopedic implants, but its clinical significance remains uncertain. While several studies report symptomatic improvement following revision with hypoallergenic implants, others describe no change or persistently poor outcomes. Such variability underscores that hypersensitivity is only one of several potential mechanisms of implant failure.

The biological plausibility of hypersensitivity is well supported: metallic ions can form haptens that trigger type IV immune reactions, resulting in chronic inflammation and osteolysis. However, not all sensitized patients develop complications, and implant failures occur in many without demonstrable hypersensitivity. This divergence pinpoints the multifactorial nature of implant survival, influenced by host, implant, and environmental factors.

Diagnosis remains a major challenge. Clinical presentations overlap with infection and mechanical loosening, while available tests – such as patch testing, lymphocyte transformation tests, and histopathology – lack standardization and predictive value. Current evidence, therefore, does not support a universal diagnostic algorithm or routine preoperative screening. A multimodal, case-by-case approach, incorporating clinical history, allergy testing, and histological findings, can ensure the best compromise between sensitivity and specificity.

From a clinical perspective, hypoallergenic implants are a reasonable option for patients with confirmed hypersensitivity or revision surgery for suspected allergy, but their widespread use cannot be justified. Preventive strategies, such as routine substitution with coated or ceramic components, have not consistently demonstrated superior outcomes.

This review has limitations, reflecting the weaknesses of the available evidence. Most studies are small, observational, and methodologically heterogeneous, limiting the ability to draw firm conclusions or perform meta-analysis. The use of a narrative synthesis could introduce a certain degree of bias, although this risk is mitigated by systematic research and selection of studies.

## Conclusion

Metal hypersensitivity is a plausible contributor to implant-related complications, but its role remains uncertain due to inconsistent diagnostic methods and heterogeneous evidence. Current data suggest considering hypersensitivity when common causes of implant failure have been ruled out and using hypoallergenic implants selectively in high-risk or revision settings. Standardized diagnostics, prospective outcome studies, and long-term evaluation of alternative materials are essential to clarify its clinical relevance and guide patient-specific management.

## ICMJE Statement of Interest

The authors declare that they have no conflicts of interest relevant to this work.

## Funding Statement

This research received no specific grant from any funding agency in the public, commercial, or not-for-profit sectors.

## Author contribution statement

VV and NM contributed equally to this work. VV was involved in conceptualization, methodology, literature search, data extraction, and drafting and editing of the manuscript. NM contributed to clinical insight, literature search, literature interpretation, and critical revision of the manuscript. TC provided clinical and dermatological expertise and performed critical revision. SS was involved in supervision, conceptualization, methodology, clinical and surgical expertise, critical revision, and final approval. All authors have read and approved the final version of the manuscript.

## References

[1] Zeng Y, Feng W, Li J, et al. A prospective study concerning the relationship between metal allergy and post-operative pain following total hip and knee arthroplasty. Int Orthop 2014 38 2231–2236. (10.1007/s00264-014-2367-1) 24910214

[2] Guenther D, Thomas P, Kendoff D, et al. Allergic reactions in arthroplasty: myth or serious problem? Int Orthop 2016 40 239–244. (10.1007/s00264-015-3001-6) 26526701

[3] Waterman AH & Schrik JJ. Allergy in hip arthroplasty. Contact Dermat 1985 13 294–301. (10.1111/j.1600-0536.1985.tb02581.x) 3912103

[4] Świeczko-Żurek B, Szumlański A & Zorn J. Allergy to implant components: chromium, nickel, cobalt and titanium. Adv Mater Sci 2009 9 41–47. (10.2478/v10077-009-0005-1)

[5] Krecisz B, Kiec-Swierczynska M & Bakowicz-Mitura K. Allergy to metals as a cause of orthopedic implant failure. Int J Occup Med Environ Health 2006 19 178–180. (10.2478/v10001-006-0025-6) 17252668

[6] Hallab N, Merritt K & Jacobs JJ. Metal sensitivity in patients with orthopaedic implants. J Bone Joint Surg Am 2001 83 428–436. (10.2106/00004623-200103000-00017) 11263649

[7] Granchi D, Cenni E, Tigani D, et al. Sensitivity to implant materials in patients with total knee arthroplasties. Biomaterials 2008 29 1494–1500. (10.1016/j.biomaterials.2007.11.038) 18155140

[8] Krecisz B, Kiec-Swierczynska M & Chomiczewska-Skora D. Allergy to orthopedic metal implants: a prospective study. Int J Occup Med Environ Health 2012 25 463–469. (10.2478/s13382-012-0029-3) 23212287

[9] Whiteside LA. Clinical results of revision TKA in patients with presumed metal and cement allergy. J Arthroplast 2022 37 (Supplement 6) S250–S257. (10.1016/j.arth.2022.02.052) 35196568

[10] Bergschmidt P, Bader R, Finze S, et al. Comparative study of clinical and radiological outcomes of unconstrained bicondylar total knee endoprostheses with anti-allergic coating. Open Orthop J 2011 5 354–360. (10.2174/1874325001105010354) PMC3195852 22016754

[11] Carlsson A & Moller H. Implantation of orthopaedic devices in patients with metal allergy. Acta Derm Venereol 1989 69 62–66. 2563611

[12] Thomas P, Weik T, Roider G, et al. Influence of surface coating on metal ion release: evaluation in patients with metal allergy. Orthopedics 2016 39 (Supplement 3) S24–S30. (10.3928/01477447-20160509-08) 27219723

[13] Dietrich K, Mazoochian F, Summer B, et al. Intolerance reactions to knee arthroplasty in patients with nickel/cobalt allergy and disappearance of symptoms after revision surgery with titanium-based endoprostheses. J Dtsch Dermatol Ges 2009 7 410–412. (10.1111/j.1610-0387.2008.06987.x) 19192161

[14] Langton DJ, Jameson SS, Joyce TJ, et al. Early failure of metal-on-metal bearings in hip resurfacing and large-diameter total hip replacement: a consequence of excess wear. J Bone Joint Surg Br 2010 92 38–46. (10.1302/0301-620X.92B1.22770) 20044676

[15] Ko JWK, Nicholson TA, Hoffler CE, et al. Metal allergy as a cause of implant failure in shoulder arthroplasty. Orthopedics 2017 40 e844–e848. (10.3928/01477447-20170719-01) 28776630

[16] Lützner J, Hartmann A, Dinnebier G, et al. Metal hypersensitivity and metal ion levels in patients with coated or uncoated total knee arthroplasty: a randomised controlled study. Int Orthop 2013 37 1925–1931. (10.1007/s00264-013-2010-6) PMC3779567 23860793

[17] Eftekhary N, Shepard N, Wiznia D, et al. Metal hypersensitivity in total joint arthroplasty. JBJS Rev 2018 6 e1. (10.2106/jbjs.rvw.17.00169) 30516716

[18] Thomas P, von der Helm C, Schopf C, et al. Periimplant histology and cytokine pattern in metal-allergic knee arthroplasty patients with improvement after revision with hypoallergenic materials. Semin Arthroplasty 2012 23 268–272. (10.1053/j.sart.2013.01.011)

[19] Thomas P, von der Helm C, Schopf C, et al. Patients with intolerance reactions to total knee replacement: combined assessment of allergy diagnostics, periprosthetic histology, and peri-implant cytokine expression pattern. Biomed Res Int 2015 2015 910156. (10.1155/2015/910156) PMC4383474 25866822

[20] Bravo D, Wagner ER, Larson DR, et al. No increased risk of knee arthroplasty failure in patients with positive skin patch testing for metal hypersensitivity: a matched cohort study. J Arthroplast 2016 31 1717–1721. (10.1016/j.arth.2016.01.024) 26869063

[21] Nam D, Li K, Riegler V, et al. Patient-reported metal allergy: a risk factor for poor outcomes after total joint arthroplasty? J Arthroplast 2016 31 1910–1915. (10.1016/j.arth.2016.02.016) 26965589

[22] Evans EM, Freeman MAR, Miller AJ, et al. Metal sensitivity as a cause of bone necrosis and loosening of the prosthesis in total joint replacement. J Bone Joint Surg Br 1974 56-B 626–642. (10.1302/0301-620x.56b4.626) 4452710

[23] Breuer R, Fiala R, Trieb K, et al. Prospective mid-term results of a completely metal-free ceramic total knee endoprosthesis: a concise follow-up of a previous report. J Arthroplast 2021 36 3161–3167. (10.1016/j.arth.2021.05.007) 34090690

[24] Atilla HA, Çevik HB, Akdoğan M, et al. Self-reported metal hypersensitivity in patients undergoing unicondylar knee arthroplasty. J Clin Orthop Trauma 2021 14 17–21. (10.1016/j.jcot.2020.10.002) PMC7919954 33680813

[25] Walker T, Rutkowski L, Innmann M, et al. Unicondylar knee arthroplasty using cobalt-chromium implants in patients with self-reported cutaneous metal hypersensitivity. Bone Joint Lett J 2019 101-B 227–232. (10.1302/0301-620x.101b2.bjj-2018-0778.r1) 30700123

[26] Beyer F, Lützner C, Kirschner S, et al. Midterm results after coated and uncoated TKA: a randomized controlled study. Orthopedics 2016 39 (Supplement 3) S13–S17. (10.3928/01477447-20160509-10) 27219721

[27] Münch HJ, Jacobsen SS, Olesen JT, et al. The association between metal allergy, total knee arthroplasty, and revision: study based on the Danish Knee Arthroplasty Register. Acta Orthop 2015 86 378–383. (10.3109/17453674.2014.999614) PMC4443448 25582229

[28] Schmidt KJ, Huang PS, Colwell CW, et al. Self-reported metal allergy and early outcomes after total knee arthroplasty. Orthopedics 2019 42 330–334. (10.3928/01477447-20190906-01) 31505017

[29] Christiansen K, Holmes K & Zilko PJ. Metal sensitivity causing loosened joint prostheses. Ann Rheum Dis 1980 39 476–480. (10.1136/ard.39.5.476) PMC1000589 7436578

[30] Pinson ML, Coop CA & Webb CN. Metal hypersensitivity in total joint arthroplasty. Ann Allergy Asthma Immunol 2014 113 131–136. (10.1016/j.anai.2014.05.012) 24934108

[31] Caicedo MS, Solver E, Coleman L, et al. Metal sensitivities among TJA patients with post-operative pain: indications for multi-metal LTT testing. J Long Term Eff Med Implants 2014 24 37–44. (10.1615/jlongtermeffmedimplants.2014010261) 24941404

[32] Willert HG, Buchhorn GH, Fayyazi A, et al. Metal-on-metal bearings and hypersensitivity in patients with artificial hip joints: a clinical and histomorphological study. J Bone Joint Surg Am 2005 87 28–36. (10.2106/00004623-200501000-00006) 15637030

[33] Milavec-Puretić V, Orlić D & Marušić A. Sensitivity to metals in 40 patients with failed hip endoprosthesis. Arch Orthop Trauma Surg 1998 117 383–386. (10.1007/s004020050272) 9709857

[34] Meyer H, Mueller T, Goldau G, et al. Corrosion at the cone/taper interface leads to failure of large-diameter metal-on-metal total hip arthroplasties. Clin Orthop Relat Res 2012 470 3101–3108. (10.1007/s11999-012-2502-5) PMC3462871 22864616

[35] Chamaon K, Barber H, Awiszus F, et al. Expression of CD11c in periprosthetic tissues from failed total hip arthroplasties. J Biomed Mater Res 2016 104 136–144. (10.1002/jbm.a.35549) 26255872

[36] Langton DJ, Joyce TJ, Jameson SS, et al. Adverse reaction to metal debris following hip resurfacing: the influence of component type, orientation and volumetric wear. J Bone Joint Surg Br 2011 93 164–171. (10.1302/0301-620X.93B2.25099) 21282753

[37] Natu S, Sidaginamale RP, Gandhi J, et al. Adverse reactions to metal debris: histopathological features of periprosthetic soft tissues following metal-on-metal hip arthroplasty. J Clin Pathol 2012 65 409–418. (10.1136/jclinpath-2011-200398) 22422805

[38] Krenn V, Morawietz L, Perino G, et al. Proposal for a histopathological consensus classification of the periprosthetic interface membrane. J Clin Pathol 2006 59 591–597. (10.1136/jcp.2005.027458) PMC1860400 16731601

[39] Krenn V, Morawietz L, Perino G, et al. Revised histopathological consensus classification of joint implant related pathology. Pathol Res Pract 2014 210 779–786. (10.1016/j.prp.2014.09.017) 25454771

